# Toward Environmental Justice in Civic Science: Youth Performance and Experience Measuring Air Pollution Using Moss as a Bio-Indicator in Industrial-Adjacent Neighborhoods

**DOI:** 10.3390/ijerph17197278

**Published:** 2020-10-05

**Authors:** Monika M. Derrien, Christopher Zuidema, Sarah Jovan, Amanda Bidwell, Weston Brinkley, Paulina López, Roseann Barnhill, Dale J. Blahna

**Affiliations:** 1 United States Department of Agriculture Forest Service, Pacific Northwest Research Station, Seattle, WA 98103, USA; dale.blahna@usda.gov; 2 Department of Environmental and Occupational Health Sciences, University of Washington School of Public Health, Seattle, WA 98195, USA; czuidema@uw.edu; 3 United States Department of Agriculture Forest Service, Pacific Northwest Research Station, Portland, OR 97205, USA; sarah.jovan@usda.gov; 4 Amanda L Bidwell, LLC, Seattle, WA 98102, USA; amanda.bidwell7@gmail.com; 5 Street Sounds Ecology, LLC, Seattle, WA 98117, USA; weston@streetsoundsecology.com; 6 Duwamish River Cleanup Coalition, Duwamish Valley Youth Corps, Seattle, WA 98108, USA; paulina@duwamishcleanup.org; 7 Duwamish Infrastructure and Restoration Training Corps, Seattle, WA 98108, USA; roseann@thedirtcorps.com

**Keywords:** environmental justice, air pollution, moss bio-indicators, citizen science, youth, community engagement, environmental education

## Abstract

This article reports on an interdisciplinary evaluation of the pilot phase of a community-driven civic science project. The project investigates the distribution of heavy metals in air pollution using moss growing on street trees as a bio-indicator in two industrial-adjacent neighborhoods in Seattle, Washington (USA). One goal of the ongoing project is to meaningfully engage local urban youths (eighth to twelfth grade) in the scientific process as civic scientists, and teach them about environmental health, environmental justice, and urban forestry concepts in a place-based, urban-oriented environmental research project. We describe the collaborative context in which our project developed, evaluate the quality of youth-collected data through analysis of replicate samples, and assess participants’ learning, career interests, and overall appraisal of the pilot. Our results indicate that youth scientists collected usable samples (with acceptable precision among repeated samples), learned project content (with statistically significant increases in scores of test-style survey questions; *p* = 0.002), and appraised their engagement favorably (with 69% of participants reporting they liked the project). We observed few changes in career interests, however. We discuss our intention to use these preliminary insights to further our community-driven education, research, and action model to address environmental injustices.

## 1. Introduction

Environmental health, environmental justice, environmental education, community-based participatory action research, youth engagement, and civic science share considerable common ground, yet are rarely addressed concurrently in the scientific literature. Their shared stake in consequential issues includes access to, and interactions with, the benefits and hazards of the environments in which people live, work, learn, and play. Environmental scholarship has long been critiqued for allowing environmental justice to remain in its blind spot [1]. Scholars have called for research on environmental health disparities to be better contextualized within their cultural, socioeconomic, and behavioral circumstances, and rooted in community processes [2,3]. Others have prompted thinking about the democratization and decolonization of the production of scientific knowledge, citing the need for affected communities to be empowered and exercise agency over adverse environmental conditions [4,5,6,7]. At the same time, civic science has shown the ability to harness the power of lay individuals to contribute to scientific discovery, though it has more often been applied to basic ecological monitoring than community-driven environmental justice research. Its success is still mainly measured in terms of data quality, rather than participant or community outcomes [8,9]. 

To meet these converging appeals, researchers strive to more meaningfully engage with communities in the definition of research problems, the development of methods, the collection and interpretation of data, and the use of results to inform mitigation actions and influence local governance contexts [10,11]. Environmental education is at once foundational and a potential outcome of meaningful engagement, across the entire spectrum of the community research-to-action process. Yet environmental education has generally failed to explicitly contextualize environmental justice issues in its curriculum, including issues that address diversity, equity, and inclusion [1]. This is especially important for communities most affected by environmental health disparities, including Black, Indigenous, immigrant, and refugee communities and communities of color. Environmental educators have been urged to transition from a unidirectional model to one of mutual learning that considers ecological topics within their sociocultural contexts [12]. In this mutual learning model, the engagement of youths is critical for success, for fostering critical thinking, problem solving, and advocacy in their communities—and not simply for creating more ecologically and socially aware citizens in the world.

We use the term “civic science” to describe the engagement of non-experts in scientific monitoring or other research activities, in acknowledgement of the problematic use of the more commonly used term “citizen science,” especially in regards to immigrant and Indigenous communities [2,13]. In our adaptation of civic science approaches in this article, we diverge from dominant paradigms that uphold an expert-centric and adult orientation in environmental research and civic science, and a unidirectional, wildland focus in environmental education. In doing so, we address issues of social and environmental justice, and their outcomes for vulnerable populations in cities. Organizing using a collective impact model approach [14], we planned and implemented an ongoing civic science project exploring the spatial distribution of air pollutants in two industrial-adjacent neighborhoods in south Seattle (Washington, USA). The project was identified, developed, implemented, and evaluated by a core collaborative group—the Green-Duwamish Learning Landscape (GDLL)—composed of the leaders of community organizations, local government officials, and government and university researchers in the health, natural, and social sciences interested in the watershed of the Green and Duwamish River, a tidal river that has been used since time immemorial by the Duwamish Tribe and more recently by immigrant communities. Known historically for its bountiful salmon, more recently the river has been marked by colonial and industrial uses, including the straightening of miles of the river to Puget Sound, and its eventual designation in 2001 as a Superfund site [15].

The core collaborative group is guided by the belief that community-based participatory action research can be used as a tool in a civic science context to address environmental injustices in three ways: (a) to share the power of knowledge creation among scientists and community members; (b) to produce data to describe the distribution of environmental hazards (or benefits) in a community; and (c) to promote actionable environmental learning for youth and adult community members, empowering them to use available networks and channels for mitigation measures. This article describes our accomplishments within aspects of all three of these dimensions.

The project engages local urban youths to collect moss samples from street trees, to be used as a bio-indicator and screening tool to identify areas with high levels of heavy metal content in particulate matter. Particulate matter (PM) is a type of air pollution composed of a mixture of solid particles and liquid droplets that is associated with increased mortality and a range of deleterious cardiovascular (e.g. heart attack and stroke) and respiratory (e.g. asthma and lung cancer) human health effects [16]. PM may contain heavy metals which are toxic on their own and add to the health risks of PM exposure [17]. PM exposures are disproportionate in many communities experiencing environmental injustices [18,19,20]. Our screening results are used to identify areas of concern within the neighborhoods, and to guide follow-up sampling and mitigation measures [21].

We explore the following interdisciplinary questions: Can trained youth scientists collect and prepare moss samples that are useable in an environmental health civic science project? Was our community-driven science approach a positive and effective forum for youth environmental learning and experiences? Did it inspire interests in environmental or community leadership opportunities? We answer these questions through an analysis of replicate moss samples, and a series of short surveys administered by leaders of the Duwamish Valley Youth Corps (DVYC). We then use these measures to inform development of curriculum and evaluation methods for project expansion.

### 1.1. Air Pollution, Environmental Justice, and Moss

Despite dramatic reductions in the United States in past decades [22], air pollution continues to harm the health of humans and the environment. Many cities still have high and unequally distributed levels of air pollution. While Seattle’s air quality is relatively good by regulatory standards [23], it does experience intra-urban variability [24]; there are local pockets or “hotspots” of pollution that are not captured by regulatory monitoring. Within the city, redlining [25] and gentrification has led to a landscape of environmental health risks that are unequally distributed, in which “riskscape and urban development burdens were skewed toward the city’s most socially vulnerable residents” [26] (p. S252), such as in the Duwamish Valley. Though long heralded for its commitment to sustainability, in recent decades Seattle has been the site of concurrent processes of environmental and industrial gentrification, during a period of rapid population growth [27,28]. The compounding of structural injustices created by these processes make clear the need for action that addresses the cumulative impacts of environmental and demographic characteristics [29].

To better understand these environmental injustices, data are needed to show the intra-urban variability of risks and how factors coincide to create these cumulative impacts. One strategy to do this for air pollution involves the analysis of pollutants accumulated in moss that grows on street trees. Moss lacks a vascular system, collecting nutrients and pollutants from the atmosphere. Moss has been used as a natural passive sampling matrix and bio-indicator of air pollution in Europe since the 1960s [30], and more recently in the United States, in Oregon [31,32] and Washington [33]. Methods have not yet been developed to relate pollutant concentrations in air to those measured in moss. While there has been considerable civic science research using networks of low-cost sensors to monitor air pollution [34,35], to our knowledge, there has been only one other use of moss chemistry as a bio-indicator of air pollution in a civic science context [36].

Collecting moss as a bio-indicator of heavy metal air pollution is well-suited for civic science because the data collection methods are easily teachable, accessible, and inexpensive compared to traditional air quality monitoring techniques that require pumps, filters, and samplers, or expensive monitoring instruments. Sample collection and preparation can be performed and guided by community members with just a few hours of fieldwork and lab training, and supervision. The simplicity of the method makes it appropriate for a “vertical mentorship” version of the “train-the-trainer” model, in which more experienced youth and adult community members informally teach newcomers about the method, increasing the size of the experienced group. Furthermore, decisions about the extent and spatial resolution of sampling can be informed by local community expertise and be collaboratively developed to reflect community interests.

### 1.2. Civic Science and Community Engagement

In civic science projects, relationships between scientists and communities are often unidimensional, with large numbers of lay people trained to systematically collect and report environmental data that cumulatively serve scientific interests, such as monitoring butterfly migrations or bird populations [37]. While long-term monitoring programs often become embedded in a large community of civic scientists, they are generally evaluated in terms of data quality and contributions, and not other dimensions of community engagement [9,10].

Observers have critiqued a dynamic in civic science in which researchers suddenly appear to conduct pre-conceived research, seeking little community engagement or input beyond individuals’ roles in collecting or providing access to needed data [6]. In contrast, researchers committed to community-driven approaches have elevated communities’ pre-existing priorities to shape scientific inquiries. For example, successful examples of environmental health research have paired community organizations with researchers for ongoing monitoring of areas of community concern; these collaborations have provided sought-after and actionable information, and fostered local champions of mitigation practices [2]. They also create the grounds for inclusion and engagement in the first place, as shown in research on participant motivation, support, and barriers to participation among historically disenfranchised community members [34]. However, this approach is not the norm in civic science, which has engaged a population that skews toward a white, higher income demographic [6,38]. While civic science is at times embedded in community-based (and -driven) participatory action research frameworks (see discussion of “extreme citizen science” [7]), it is more often situated in civic science’s contributory realm [6]. Nevertheless—and in all of these realms—civic science offers important opportunities to meaningfully engage communities in participatory research and change the “relationship between science, expert knowledge and citizens in democratic societies” [4] (p. 24).

### 1.3. Environmental Education Foundations for Civic Science in Cities

Civic science contexts can help promote environmental health literacy among youths and create connections with environmental research and community action [39]. Both formal and informal environmental education makes meaningful participation in civic science possible [9]. The U.S. Environmental Protection Agency’s definition of environmental education focuses on problem solving and action, supported by basic environmental learning [40]. While this focus lends support for the GDLL’s integrated approach, our approach bucks a dominant trend in environmental education by explicitly framing educational material within environmental justice concepts [1,41].

Furthermore, with our urban-centric approach, we diverge from a paradigm in the United States in which environmental education predominantly occurs in (and focuses on) environments outside of cities [41]. Environmental education in cities can help dispel the notion that the “nature” that matters is outside of cities and improve understandings of natural processes and people’s relationships to them [42]. It can also be helpful for fostering senses of place among urban youths [42,43]. Many popular civic science projects monitor species and phenomena within people’s immediate surroundings [44,45]. However, scholars have observed that environmental learning needs to be expanded beyond its narrow biophysical focus in environmental education, to equip people to meaningfully examine social-ecological systems, asking questions such as “Who determines what happens here? At what cost? To whose benefit? Why not somewhere else?” [12] (p. 38).

Civic science projects, though typically geared towards adults, have been shown to be engaging and effective in generating positive outcomes for youths [46,47]. The pairing of youth-centered civic science projects with environmental education has mutually reinforcing outcomes, especially for youths from racial and ethnic groups underrepresented in science. In science classroom settings, project- and inquiry-based approaches have shown positive effects on student achievement among students from underrepresented groups [48]. Working with communities to develop projects that serve immediate needs promotes both engagement and relevancy [6]. As a result, community-driven civic science offers the opportunity—one that environmental education often misses—to contextualize environmental learning for youths within concepts of justice and equity [1,41]. 

In summary, our project introduces a community-driven civic science approach that builds on established biomonitoring methods to advance needs identified in the environmental health, justice, and education literatures to engage urban youth and adult community members in scientific inquiries. We designed our pilot to reveal insights into how we can ensure data quality and provide positive and enriching experiences as the project advances.

## 2. Materials and Methods

To implement the project, the GDLL core collaborative group identified and convened key project participants from the United States Department of Agriculture Forest Service’s Pacific Northwest and Northern Research Stations, the Seattle Office of Sustainability and Environment, the Duwamish River Cleanup Coalition (DRCC), the Duwamish Infrastructure and Restoration Training Corps (DIRT Corps), Western Washington University, the University of Washington, Street Sounds Ecology, and Just Health Action. Informed by Pandya’s framework for inclusive design [49], the group identified the overall study hypotheses and methods, and developed the curriculum for youth civic scientists. By combining field investigation with primary data collection and project-based learning, our approach integrates what Stern, Powell, and Hill describe as the three most effective environmental education pedagogies [50].

### 2.1. Study Area

**Figure 1:**
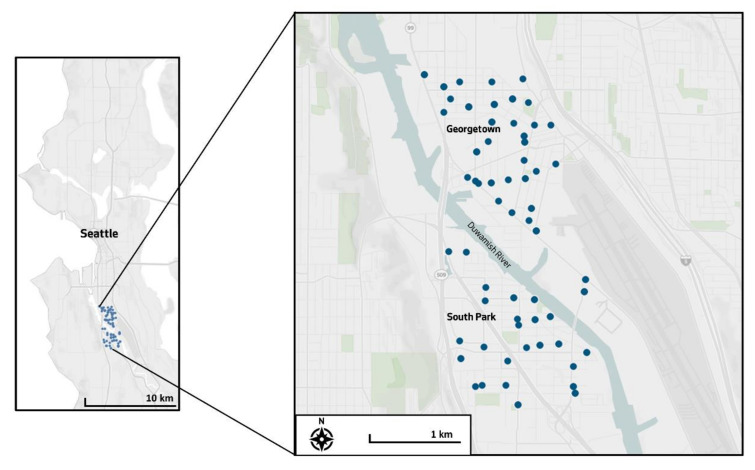
Map of the study area including the neighborhoods of Georgetown and South Park in Seattle, WA. Circles indicate moss sampling locations.

The pilot phase of our project took place in Georgetown and South Park, two neighborhoods in south Seattle that are located within a major transportation corridor, near interstate highways, the Port of Seattle, a major rail line, and an airport (Figure 1). Other industrial activities, including manufacturing and scrap metal and glass recycling, are in close proximity to residences. The Duwamish River flows between the two neighborhoods, part of a five-mile stretch designated as a Superfund site because of its contamination with numerous hazardous industrial chemicals including polychlorinated biphenyls (PCBs), arsenic, polycyclic aromatic hydrocarbons (PAHs), and dioxins [15]. As is the case in many urban, lower income neighborhoods, residents face disproportionate socioeconomic, health, and environmental challenges. There is a growing body of evidence that Georgetown and South Park are burdened with some of the worst health and air pollution disparities in the region [51,52,53]. Washington’s Environmental Health Disparities map shows that neighborhoods in the study area have the highest ranks of environmental health disparities, environmental exposures, and PM concentrations in the state [54,55]. These neighborhoods, often referred to as “environmental justice” communities, are mostly within the 98108 ZIP code, where 63.9% of residents are nonwhite, 34.7% are foreign-born, 19.4% live below the poverty level, and less than one third hold a bachelor’s degree or higher [56]. 

### 2.2. Duwamish Valley Youth Corps Context and Pilot Participants

The pilot’s youth scientists were all members of a paid ten-week program with DRCC’s DVYC, and nearly all identified as people of color from Georgetown and South Park. They were recruited through a network of community outreach including teachers, environmental educators, and other community leaders. DVYC promotes a culture of mentorship and teamwork for local youths, offering experience and training for environmental career paths, and engaging youths in outdoor projects that explore the connection between the natural world and human activity in the Duwamish Valley. Prior to their engagement in the pilot, participants learned about patterns in environmental quality and ways to improve conditions through urban forestry and green infrastructure, including modules on the benefits and economics of urban trees, the links between transportation, industry and pollution, and river restoration. DVYC projects put this knowledge into action through activities in local neighborhoods such as youth-designed and -led tree giveaways, green wall and rain garden projects, and nearshore river restoration using native plants. These prior projects prepared youths for the pilot with scientific skills and fluency in environmental justice topics.

The pilot took place on eight days over the course of one month (mid-May to mid-June 2019), as the second portion of the DVYC program. During the pilot, 26 participants joined twice-weekly meetings, for one three-hour weekday afternoon session, and one four-hour weekend morning session. Participants were 8th–12th graders, with most in 8th or 9th grade, attending schools in Seattle, Burien, White Center, and SeaTac (Table 1). At the beginning of the project, participants listed their favorite subjects in school as math (63%), science (47%), English/writing (37%), history (32%), and other subjects.

### 2.3. Pilot Locations, Structure, and Personnel

Pilot activities were convened at local community centers, on neighborhood streets, and in a high school science lab. Activities included two indoor sessions to learn about the project and methods, four outdoor sample collection sessions in South Park and Georgetown, and two sample preparation sessions at a high school science lab. These activities were led by scientists and community leaders who were part of the core collaborative group; at least four adult project partners engaged with participants at each session, delivering background content, participating in small-group sampling teams, and advising on youth work in the science lab. 

### 2.4. Moss Data Collection, Preparation, and Analysis

Sampling and preparation methods were adapted from earlier work by professional scientists [32], with minor changes in presentation and procedures to adapt protocols for youth participants. On sample collection days, the participants worked in groups of three to five people, collecting moss samples guided by a quarter kilometer sampling grid (250 m × 250 m) across the study area (Figure 1). Participants used a map application on smartphones to navigate to the centroids of pre-assigned grid cells, and visually identified the nearest accessible tree. The teams then assessed the tree for the target species, Lyell’s bristle moss (*Orthotrichum lyellii* Hook. & Taylor). If no moss was present, the team went to the next nearest tree from the centroid. If no tree with sufficient moss was found within the block, or if conditions did not allow access, participants moved on to the next grid cell. At a target of 20% of grid cells, youth scientists immediately collected a replicate moss sample. The purpose of youth replicates was to assess the precision (also referred to as repeatability or reliability) of the sampling methods (i.e., if metal concentrations were the same for repeated samples). In addition, professional “expert” scientists who had been involved in similar studies re-sampled a target of 20% of grid cells following the youth sampling sessions, with the majority of expert replicates collected 12 days after the youth samples. The purpose of expert replicates was to assess the accuracy of youth samples (i.e., if metal concentrations were the same in youth and expert samples). The use of replicates in environmental sampling is a well-established technique to analyze and characterize sources of variability and error [57,58,59]. 

In the sample preparation sessions, participants individually prepared the moss samples in the lab. They wore non-powdered nitrile gloves, and cleaned their workspace and tools before and after each sample. Preparation involved harvesting the upper two thirds of living moss stems and using forceps to remove foreign debris, to produce samples that were at least 1.5 grams. The expert scientists prepared their samples using the same methods. Prepared moss samples were mailed to an analytical chemistry lab to quantify the concentration of 25 elements using inductively coupled plasma optical emission spectrometry (ICP-OES) [32]. Further analysis and interpretation of these lab results focused on six heavy metals commonly associated with negative human health and environmental effects: arsenic (As), cadmium (Cd), cobalt (Co), chromium (Cr), nickel (Ni), and lead (Pb) (referred to hereafter as “priority metals”). The metal concentrations in moss reported here cannot be directly translated to metal concentrations in air.

We used a variety of graphical and statistical procedures to assess the agreement between primary youth samples and replicates and primary youth samples and expert replicates. We prepared figures displaying paired differences between samples and replicates with “bounds of agreement” at ±1 and ±2 standard deviations about the mean difference. We conducted a series of parametric (t-tests) and non-parametric (Kolmogorov–Smirnov tests) to compare youth samples to replicates. We also used linear regression to compare moss metal concentrations in youth samples and replicates summarizing the regression intercept, slope, R^2^, and root mean square error (RMSE).

### 2.5. Survey Data Collection and Analysis

Because the primary objective of this pilot phase was to assess if participants could adequately collect and prepare moss samples, the short surveys for this pilot were a secondary focus designed to inform the development of future project evaluations. All participants and their custodians (for minors) gave their informed consent for inclusion before they participated in the DVYC program and evaluation, as approved by the DRCC Board of Directors. Participants completed survey components administered on four pilot days that contained a different combination of test-style and survey-style questions (Table 2). The components included questions about: academic interests (one open-ended question); career interests (one open-ended and one Likert-scaled question with 16 sub-questions); moss and urban forestry (five test-style questions); moss sampling methods (six test-style questions); moss sample preparation (six test-style questions); and likes and dislikes about the pilot (two open-ended, and one Likert-scaled question). We dropped one question from the moss sample preparation set because of unclear wording. Survey components b through e were administered twice (Table 2): career interest questions (component b), were administered at the first and last session, and test-style questions (components c through e) were administered before and after relevant project content was taught. Out of 26 participants, between 10 and 16 participants completed individual survey components that were administered twice (hereafter referred to as “pre-post pairs”). Nine participants completed all survey components.

The questions in components a, b, and f (Table 2) had no “right” and “wrong” responses. For these, we have presented descriptive statistics to summarize the data. The questions in components c, d, and e had pre-determined “right” and “wrong” responses. We performed t-tests, paired, where appropriate, for survey components that measured pre- and post-youth learning and career interests. 

## 3. Results

We first present our findings on youth performance from our replicate design to measure data quality. We then present our findings on youth experiences from our surveys.

### 3.1. Youth Performance

We discarded 15% of youth moss samples because two were the wrong species, and 13 were incorrectly labeled. The final analytical dataset had 79 youth samples from 61 locations; 18 of these samples were youth replicates. Expert scientists collected an additional 20 samples as expert replicates, three of which we did not use in our analysis because their student pair was discarded.

**Figure 2:**
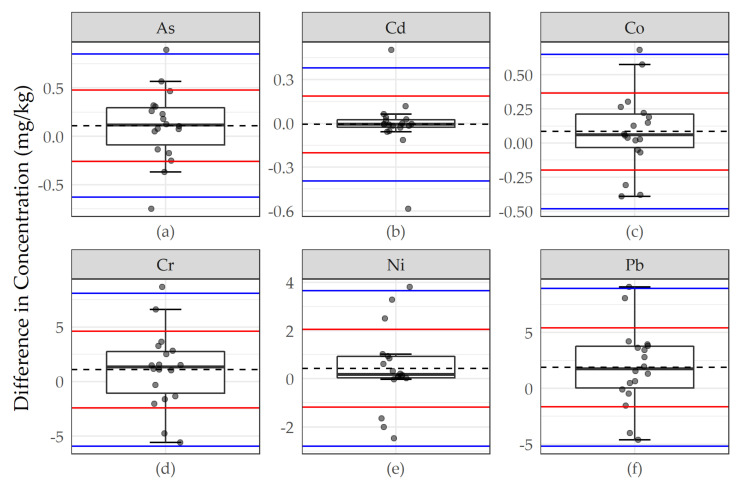
Differences in concentration in youth scientists’ primary and replicate moss samples for (**a**) As, (**b**) Cd, (**c**) Co, (**d**) Cr, (**e**) Ni, and (**f**) Pb. Points represent paired differences, summarized by underlying boxplots. Dashed lines represent the mean paired difference between primary and replicate samples; red and blue lines represent “bounds of agreement” at ±1 and ±2 standard deviations, respectively, about the mean difference. Arsenic (As), cadmium (Cd), cobalt (Co), chromium (Cr), nickel (Ni), and lead (Pb).

Youth moss samples were generally repeatable. Differences in metal concentrations (mg/kg) between replicate pairs were small, with most falling within ±1 standard deviation (SD) of the mean difference and distributed about a difference of zero (Figure 2). We observed a statistically significant difference between paired primary and replicate samples for only Pb among the six priority metals with a paired t-test (*p* = 0.04). Using a non-parametric test (Kolmogorov–Smirnov test), there were no statistically significant differences between the youth scientists’ primary and replicate distributions. 

**Figure 3:**
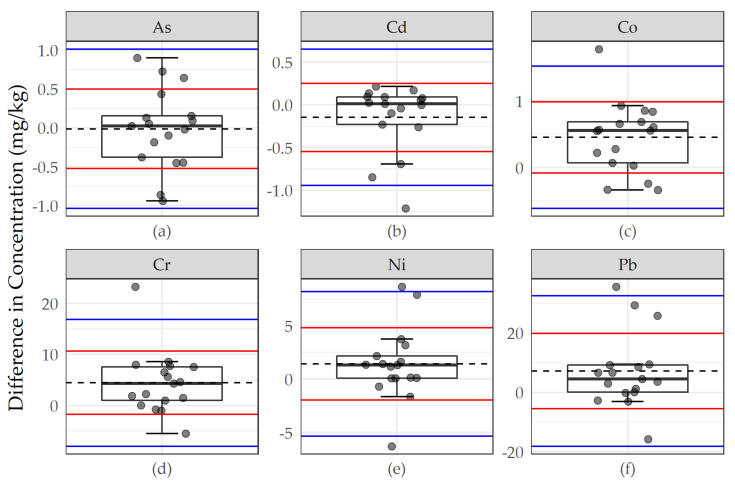
Differences in concentration between youth scientists and expert replicates for (**a**) As, (**b**) Cd, (**c**) Co, (**d**), Cr, (**e**) Ni, and (**f**) Pb. Points represent paired differences, summarized by underlying boxplots. Dashed lines represent the mean paired difference between youth primary and expert replicate samples; red and blue lines represent “bounds of agreement” at ±1 and ±2 standard deviations, respectively, about the mean difference.

Differences between youth primary moss samples and expert replicate pairs were greater than those observed between youth primary moss samples and youth replicate pairs, with metal concentrations in youth moss samples tending to be higher (Figure 3). Table 3 summarizes the moss metal concentrations for all primary youth samples, the mean differences for youth primary and replicate samples, and the mean differences between primary youth and expert replicate samples. The differences between youth samples and expert replicates were less than the mean concentration and standard deviation observed among youth samples for each of the priority metals. Among the metals where differences were normally distributed (As, Co, Ni and Pb), we observed statistically significant differences for Co and Pb with paired t-tests (*p* ≤ 0.05). Those differences that were statistically significant with t-tests, however, as well as the differences for all other metals, were not present with the Kolmogorov–Smirnov test.

The results of linear regressions comparing primary youth samples and youth replicates, and primary youth samples and expert replicates are shown in Table 4. The youth samples and replicates exhibit good agreement with regression slopes near one, intercepts near zero, R^2^ ≥ 0.70 and RMSE less than both the mean and standard deviation of the metal concentration among primary youth samples. The performance measures summarizing the regression comparison between youth primary samples and expert replicates were lower, however. Slopes were further from one, intercepts were further from zero, R^2^ were lower, and RMSEs were greater than the primary youth samples and youth replicates.

### 3.2. Youth Experiences

#### 3.2.1. Learning Outcomes

DVYC surveys showed that participants learned key project content over the course of the pilot (Table 5), with improved performance on all test-style survey components. Overall, pilot participants demonstrated improvements in test scores for the three content-focused survey components, although only the improvements for the “moss sampling methods” component neared significance for the unpaired comparisons. For the comparison of pre-post pairs, participants also showed improvements for the three content-focused components, although only the improvements for the “moss, air pollution, and urban forestry” and “moss sample preparation” were significant. For the nine participants who completed all survey components at every session (all pre-post pairs), there was a statistically significant improvement of 9.3% in overall scores. 

We considered differences in youth learning between those who listed science as one of their favorite subjects in school, and those who did not list science as one of their favorite subjects in school. The test scores for these two groups showed no statistically significant difference. We also considered differences between youth learning for different types of questions, comparing questions that were based on content only taught in the classroom, and questions that were taught in the classroom and reinforced through hands-on application during sample collection and preparation activities (field and lab work). There were no statistically significant differences between these types of test questions.

#### 3.2.2. Career Interests

Participants were asked about their career interests at the beginning and end of the pilot, on a Likert scale from “not at all interested” (1) to “very interested” (5). Table 6 shows that career interests generally did not change much over our time period. A priori, we thought that participants’ interests in “forests, parks, nature,” “community organizing or politics,” “air or water pollution,” “city/community planning,” and “science/research” might increase because of our activities, but we did not observe statistically significant increases in these (or any) job fields. Our results do suggest there was an increase in interest in “health care/medicine,” and “food/beverage services” and a decrease in “animal care, veterinary,” though the results were not statistically significant. Three out of four job fields in which participants were most interested at the beginning and end of the pilot were the same: “sports, athletics,” “crime investigation/forensics,” and “music, art, entertainment.” 

#### 3.2.3. Appraisal of Experience

At the end of the pilot, participants were asked if they liked or disliked the project, compared to other DVYC projects in which they had been engaged. Responses were generally favorable. Of the 19 participants who responded, 16% said they “liked it a lot”, more than half (53%) of participants said they liked the project “a little”, about a quarter (26%) of participants said the project was “OK”, and only one participant (5%) gave the project a negative appraisal (“disliked it a little”). None “disliked it a lot”. We had data on favorite school subjects for 16 of these 19 participants; a higher proportion of participants who favored science liked the project “a little” or “a lot” (6/7; 85.8%) compared to those who did not favor science (5/9; 55.6%). 

Participants were also asked two open-ended questions about what they liked and did not like about the project. These were categorized. For the 19 participants who responded, the most common responses about what they liked were: being outside (8 mentions), working with moss (5), the teamwork aspect (4), and learning new things (4). The most common responses to what they did not like were: preparing moss samples in the lab (5 mentions), the extent of walking involved in sampling (5), and when they had difficulty finding moss (4). 

## 4. Discussion

Our project is fueled by the need for education, engagement, and action to redress environmental injustices, and the importance of including youths in meaningful and consequential environmental research to inform these processes. This article details our measures of youth performance and experience in our project’s pilot phase. Our results indicate that the concentrations of priority metals in youth-collected moss samples were generally precise, with results of graphical and statistical procedures in agreement on the repeatability of youth samples. The strength of agreement between youth samples and expert replicates, however, was less clear, with mixed evidence among our graphical and statistical analyses. The differences between youth samples and expert replicates, though potentially statistically different, may not be meaningfully different compared to the average metal concentrations and variability observed among primary youth samples. Despite the differences, we were still able to use the youth moss samples in this pilot in subsequent analyses to provide information about the spatial variability in moss metal concentrations in the study area. A previous civic science project using the moss method avoided having a group of undergraduate students prepare the moss samples they had collected, out of concern for sample contamination [36]. Our experience demonstrates that by providing the training, environment, and guidance needed to prepare moss samples, even secondary school students can be successful and remain engaged in all phases of primary data preparation. Our results also demonstrate that youth participants learned project content over their five-week involvement in the pilot, were generally positive about their experiences, and did not exhibit short-term changes in career interests. Participants who favored science as a subject in school appraised the project more favorably than those who did not favor science. 

Our insights from the pilot will inform the continued development of project protocols and integrated evaluations of youth experiences and data quality. Given our success with the quality of youth sampling, we will proceed to expand our approach, increasing the number of youth scientists and organizations who are engaged, increasing the spatial extent of moss sampling, and creating more robust program curriculum and evaluation. Because this pilot was implemented by a team working together for the first time (and adjusting established protocols for youth civic scientists), we managed our process adaptively. Our timing was not always ideal for youth engagement. For example, the laboratory and data analysis took longer than expected, so participants did not learn about their results for many months following data collection. It is notable, however, that despite the coronavirus pandemic, 18 youth corps members chose to attend a (physically distant) results sharing event in August 2020 that was over a year after their initial involvement in data collection. This was followed by a three-week DVYC program that was attended by 15 of the pilot’s original participants and focused on environmental justice and health disparities. The group mapped the moss results to guide considerations of potential neighborhood sources. Following the program, three youth participants volunteered to present the data and their interpretation of the results at the fall meeting of the Lower Duwamish Clean Air Task Force—a group of 40–50 air quality experts and community stakeholders. In the future, when we have a faster pace with refined processes, we hope to improve the continuity of youth experiences by engaging them in subsequent phases—interpreting results, sharing results with communities, and initiating mitigation actions—with less of a gap in timing. This is important for maintaining enthusiasm, extending learning, and retaining participants. It may also allow for more youth leadership in project implementation, reducing the extent of adult involvement at many phases. 

We also gained several insights for refinements to our sampling methods. In the future, we will simplify and emphasize the importance of labeling procedures and ensure all participants receive appropriate training and practice with sample labeling to avoid discarding otherwise viable samples. Through this pilot we also learned how important it is to collect replicate moss samples as near in time as possible to primary samples. Logistical constraints prevented experts from collecting replicate samples immediately following youth sample collection. The nearly two-week delay may explain some of the differences in metal concentrations between youth samples and expert replicates. While for this reason, the pilot’s expert replicates may not have provided an ideal comparison to the youth samples to characterize differences attributable to sampling and preparation, they may provide some insight into temporal variability of moss metal concentrations. Environmental factors during that 12-day delay, such as precipitation events, may be responsible for the differences observed between youth and expert replicate samples. Identifying these important environmental factors related to variability in moss metal concentrations is the subject of future analyses. Additionally, we will add expert replicate samples to the sampling design so that we have expert–expert replicates in addition to our youth–youth replicates and youth–expert replicates. These changes will reduce potential factors leading to disagreement between youth and expert samples and improve our ability to identify and quantify sources of systematic bias in our methods. 

One of our challenges in assessing experiences in the pilot was the variability in youth attendance over our eight moss-focused sessions. In the DVYC program design, participants have a required number of sessions for successful completion of the program, but those who miss a session can make it up at a later date, which could be focused on a different project. As a result, the size and composition of our youth scientist population varied by session. Of the total 26 participants who engaged in various sessions of the moss pilot, only 9 had full attendance. All community-based civic science projects will have some unpredictability due to the voluntary nature of participation, making it important to understand how to work within structures to ensure successful project implementation. Regular attendance has added importance in civic science projects because it is often related to the quality of data collected (i.e., the learning curve effect) [37]. Our pilot design was built on a full cycle of learning, training, collection, and follow-up sharing and actions, but our youth scientists had many competing work, school, and family demands, and could not conform to overly rigid schedules. This makes our pilot’s data quality and modest learning findings even more notable for an environmental education and civic science model. This also suggests the likelihood for success for projects using our moss methods in settings that may be structurally more conducive to consistent attendance, such as within formal learning settings like high school or college classes or internships. 

Results of this pilot suggest future evaluations with larger sample sizes, more sophisticated measures, and longer and more consistent engagement will demonstrate even more promising outcomes for youths. As we advance to a larger project scale in our next phase, we will be able to improve the evaluation model in several ways. First, we will engage high school science club members and evaluate their experience in comparison to a control group of peers who were not involved in the project to better understand the effects of the program among participants on learning, career interests, and other measures. Second, we will evaluate the relationship between experience level and data quality, to understand how the length and consistency of engagement is related to the development of civic science skills. Third, we will add new measures to incorporate metrics for environmental literacy and personal and civic responsibility, including participants’ agency, self-efficacy, and behavior (as described by [60,61,62,63,64]). Finally, we will introduce more open-ended interview- and journal-style components, to learn how participants frame their experiences in their own words. 

These approaches will be implemented during multiple phases of the project, including measures that extend to subsequent project phases (interpretation, sharing, mitigation), to capture the trajectory of longer term and multi-faceted learning. For example, one 15-year old participant shared with a program organizer after the pilot:

*“That gave me another view on how bad the pollution is and hurting the environment, but also ourselves! This opened my eyes more and showed me that we as humans need to help even if it’s just learning about moss and how it catches the pollution around. But my biggest takeaway from the program was to tell my friends and family about taking care of our earth, and making it a better sustainable environment.”*

These sorts of reflections offer important insights into how youths perceive and are affected by their engagement, and in our future design, our evaluation will capture these systematically. 

Our project benefits from a co-production model of science and the integration of approaches from environmental education, environmental justice, environmental science, and environmental health. The model also comes with the conflict and challenges of collaborative, interdisciplinary, and cross-sector work, as well as a less centralized, university-centric approach than other collaboratives have used [65]. To better understand this part of our process, we also plan to assess adult project partners’ experiences in the multiple layers and iterations of the project, to understand if and how youth learning was supported by mutual learning among community leaders and scientists at key project phases. This will likely include some of the measures described earlier (environmental literacy and personal and civic responsibility), collected through interviews and surveys. 

Finally, in retrospect, our finding that career interests did not change over the course of the five-week period was not surprising. We would, however, hope to see effects with longer term or more consistent engagement in the project. Age may have played a factor here, too: our participants, most of whom were in late middle school and early high school, may not have been thinking seriously about career options yet, even if they were exploring and developing interests and skills in a less career-oriented way. Future inquiries about careers should be considered through this age-specific lens. Furthermore, in assessing career or more general environmental interests, we expect to be faced with some of the same challenges in generating change that other such science programs face [48]. Students’ families may perceive environmental fields to be less desirable than other sectors, contributing to the “participation problem” for environmental careers. Trends in narrowly focused environmental education may lead students, especially those from cities, low-income households, or ethnic or racial minorities, to perceive environmental issues as outside of their realm of existence [6,66]. We observed these patterns with the pre- and post-self-reporting of participants’ career interests (Table 6); even for youths engaged in a voluntary environmental program, baseline career interests in environment-oriented careers were low. This is even though participants described the strongest interests in science and math as subjects in school. We see our community-driven civic science approach not only as an important tool for advancing youth involvement in environmental research in cities, but also for more broadly fostering engagement in a more diverse environmental movement. 

## 5. Conclusions

Our findings suggest that community-driven civic science can be an important forum for engaging youths in environmental justice and health concepts as well as collecting relevant data to inform action. In the project’s pilot phase, we documented youth learning, positive experiences, and the ability of youth scientists to collect primary scientific data; these findings will inform the next phase of our project’s development. Not all community-based research requires intensive engagement during all phases, but those that do have “the additional benefits to both researchers and impacted communities through information sharing, training and research experiences, empowerment of impacted communities, improved recruitment of participants and quality of collected data, and increased research capacity” [2] (p. 8). While these intended outcomes were central to our efforts, we also tried to use them to build momentum within a diverse environmental movement, that speaks to the multiple dimensions of environmental issues that may be differentially salient to groups [6]. Civic science’s ability to engage underrepresented groups is critical and challenging to its relevancy and impact. By delivering environmental education through community-driven civic science projects, our intention is to co-produce actionable science to redress and mitigate environmental health injustices in Lower Duwamish communities; our pilot evaluation has given us important insights for our continued work toward this goal. 

## Figures and Tables

**Table 1:** Pilot participant characteristics.

Participant Characteristics	Count
Total pilot participants	26
8th graders	7
9th graders	10
10th–12th graders	2
Number of schools represented	5
Missing school and grade information	7

**Table 2:** Number of youth participants completing survey components during pilot sessions.

Survey component	Session 1	Session 2	Session 3	Session 4	Pre-post Pairs
a. Favorite academic subjects	19	-	-	-	-
b. Career interests	19	-	-	19	16
c. Moss, air pollution, and urban forestry	20	17	-	-	13
d. Moss sampling methods	-	17	15	-	10
e. Moss sample preparation	-	-	15	19	14
f. Appraisal of experience	-	-	-	19	-

**Table 3:** Summary of mean metal concentration for youth primary moss samples, mean difference between youth primary and replicate samples and mean difference between youth primary, and expert replicate samples for each priority metal.

Metal	Youth Primary Sample Mean Concentration ± SD (mg/kg) (*n* = 61)	Youth–Youth Mean Difference ± SD (mg/kg) (*n* = 18)	Youth–Expert Mean Difference ± SD (mg/kg) (*n* = 17)
As	1.184 ± 0.642	0.110 ± 0.370	−0.013 ± 0.513
Cd	0.560 ± 0.386	−0.007 ± 0.194	−0.147 ± 0.340
Co	1.827 ± 1.738	0.084 ± 0.283	0.453 ± 0.534
Cr	16.835 ± 12.722	1.096 ± 3.500	4.449 ± 6.204
Ni	7.937 ± 7.902	0.430 ± 1.613	1.435 ± 3.384
Pb	23.860 ± 18.731	1.893 ± 3.525	7.195 ± 12.663

**Table 4:** Summary measures of linear regressions comparing primary youth moss samples with youth replicates, and primary youth moss samples with expert replicates.

	Youth–Youth (*n* = 18)				Youth–Expert (*n* = 17)			
Metal	Slope	Intercept (mg/kg)	R^2^	RMSE ^1^ (mg/kg)	Slope	Intercept (mg/kg)	R^2^	RMSE (mg/kg)
As	1.11	−0.23	0.74	0.35	0.89	0.10	0.32	0.50
Cd	1.11	−0.04	0.86	0.18	0.48	0.19	0.36	0.30
Co	1.03	−0.12	0.85	0.27	1.08	0.38	0.46	0.52
Cr	0.94	−0.28	0.82	3.37	0.95	4.97	0.40	6.01
Ni	0.72	1.26	0.70	1.35	0.31	4.78	0.09	2.70
Pb	0.95	−1.10	0.87	3.40	1.54	−1.48	0.59	11.34

^1^ Root mean square error.

**Table 5:** Scores on test-style survey components in the 1st and 2nd rounds of administration, stratified by unpaired and paired comparisons.

Survey Component ^1^	1st Round Mean ± SD (*n*)	2nd Round Mean ± SD (*n*)	Mean Difference	t-test *p*-value
All Participants, Unpaired				
c. Moss, air pollution, and urban forestry	78.3 ± 17.0% (*n* = 20)	85.3 ± 14.0% (*n* = 17)	7.0%	0.181
d. Moss sampling methods	65.7 ± 17.4% (*n* = 17)	78.0 ± 17.0% (*n* = 15)	12.3%	0.053
e. Moss sample preparation	61.1 ± 25.5% (*n* = 15)	77.1 ± 20.7% (*n* = 19)	18.8%	0.060
Total ^2^	73.3 ± 14.1% (*n* = 9)	82.6 ± 11.2% (*n* = 10)	9.3%	0.100
Pre-Post Pairs				
c. Moss, air pollution, and urban forestry	73.6 ± 17.5% (*n* = 13)	87.4 ± 14.0% (*n* = 13)	13.8%	0.012 *
d. Moss sampling methods	72.7 ± 8.1% (*n* = 10)	79.0 ± 13.2% (*n* = 10)	6.3%	0.213
e. Moss sample preparation	61.1 ± 26.5% (*n* = 14)	82.0 ± 18.9% (*n* = 14)	20.9%	0.002 *
Total ^3^	73.3 ± 14.1% (*n* = 9)	82.6 ± 11.9% (*n* = 9)	9.3%	0.002 *

^1^ These include the scored survey components lettered in Table 2. ^2^ This total refers to participants’ scores who completed all three survey components from the 1st or 2nd rounds of survey component administration (unpaired). ^3^ This total refers to participants’ scores who completed all three survey components from the 1st and 2nd rounds of survey component administration (paired). * *p*-values ≤ 0.05 are considered statistically significant.

**Table 6:** Participants’ levels of interest in future jobs fields (paired pre-post surveys; *n* = 16).

Future Job Fields	1st Round Mean ± SD	2nd Round Mean ± SD	Mean Difference	t-test *p*-value
Health care/medicine	2.6 ± 1.6	3.2 ± 1.2	0.6	0.055
Police/fire	1.9 ± 1.3	2.4 ± 1.1	0.5	0.164
Teaching/education	1.8 ± 1.1	2.3 ± 0.9	0.5	0.104
Food/beverages services	1.8 ± 1.1	2.3 ± 0.9	0.5	0.070
Forests, parks, nature	2.4 ± 1.5	2.8 ± 1.1	0.4	0.289
Music, art, entertainment	3.0 ± 1.7	3.3 ± 1.4	0.3	0.464
Landscaping or lawn/garden care	1.8 ± 1.2	2.0 ± 1.0	0.2	0.549
Mechanical or electrical trades	2.1 ± 1.2	2.3 ± 1.4	0.2	0.523
Community organizing or politics	1.8 ± 1.2	1.9 ± 1.2	0.1	0.270
City/community planning	2.0 ±1.0	2.0 ± 1.4	0.0	1.000
Sports, athletics	3.4 ± 1.2	3.4 ± 1.2	0.0	0.669
Wildlife/fish protection	2.4 ± 1.5	2.3 ± 1.0	−0.1	0.637
Science/research	2.5 ± 1.4	2.4 ± 1.0	−0.1	0.697
Air or water pollution	2.6 ± 1.5	2.4 ± 1.2	−0.2	0.580
Crime investigation/forensics	3.2 ± 1.6	3.0 ± 1.1	−0.2	0.682
Animal care, veterinary	3.3 ± 1.2	2.7 ± 1.1	−0.6	0.083
